# A Separated Reset Waveform Design for Suppressing Oil Backflow in Active Matrix Electrowetting Displays

**DOI:** 10.3390/mi12050491

**Published:** 2021-04-27

**Authors:** Linwei Liu, Pengfei Bai, Zichuan Yi, Guofu Zhou

**Affiliations:** 1 Guangdong Provincial Key Laboratory of Optical Information Materials and Technology & Institute of Electronic Paper Displays, South China Academy of Advanced Optoelectronics, South China Normal University, Guangzhou 510006, China; linwei.liu@guohua-oet.com (L.L.); guofu.zhou@m.scnu.edu.cn (G.Z.); 2 College of Electron and Information, University of Electronic Science and Technology of China, Zhongshan Institute, Zhongshan 528402, China; yizichuan@zsc.edu.cn; 3 Academy of Shenzhen Guohua Optoelectronics, Shenzhen 518110, China

**Keywords:** electrowetting display (EWD), active matrix, oil backflow, dynamic reset waveform, field programmable logic array (FPGA), driving waveform

## Abstract

The electrowetting display (EWD) is a kind of reflective paper-like display. Flicker and grayscale distortion are caused by oil backflow, which is one of the important factors restricting the wide application of EWDs. The charge embedding caused by the electric field force in the dielectric layer is the cause of oil backflow. To suppress oil backflow, a separated reset waveform based on the study of oil movement is proposed in this paper. The driving waveform is divided into two parts: a reset waveform and a grayscale waveform. The reset waveform generated by a reset circuit can be used to output various voltages. The grayscale waveform is set as a traditional PWM waveform. The reset waveform is composed of a charge-releasing stage and oil-moving back stage. Two phases are contained in the charge releasing stage. The overdriving voltage is used during the first phase to reverse the voltage of all pixels. The trapped charges can then be released from the dielectric layer during the second phase. A higher voltage is used during the oil-moving back stage to drive the oil faster in the pixel. By comparing the experimental data, the oil backflow time is extended 761 times by the reset waveform. The four grayscales can be maintained by the reset waveform after driving for 300 s.

## 1. Introduction

The liquid crystal display (LCD) and organic light emitting diode (OLED) have become indispensable parts of information interactions [1,2,3]. However, the advantages of reflective displays are still irreplaceable by LCDs [4]. Currently, electrophoretic display (EPD) technology is widely used in the fields of electronic books, electronic labels, and smart watches [5,6,7]. Electronic books, represented by Amazon (Seattle, American) and iFlytek (Hefei, China), are successfully commercialized reflective display products [8,9,10]. Ghost images and flickering when reading e-books can cause eye discomfort. In addition, when playing videos on commercial EPDs, it is difficult to ensure smooth display [11,12,13,14].

The EWD technology also belongs to the reflective display technologies. Its characteristics are similar to those of EPDs, including paper-like reading and low power consumption, but its response speed is faster. In 2003, an EWD based on the characteristics of electrowetting was designed and published in *Nature* by Hays [15]. The main structure of the EWD has been adopted up to the present. Its response time is less than 10 ms, which is suitable for playing videos [16]. As a reflective display with fast response speed, it has attracted many scholars and investors, promoting its research and development. The characteristics of low power consumption [17,18], high reflectivity [19], grayscale [20,21], and color display of EWDs were gradually reported by researchers [22].

However, the advancing and receding contact angles of oil in an EWD pixel are different, which shows the phenomenon of hysteresis. In addition, the charge trapping in the dielectric layer of the EWD pixel shows the phenomenon of oil backflow, which have a negative effect on optical properties. The charge trapping and hysteresis phenomena in EWDs increase the complexity of displaying grayscales. In order to release the charge trapping and reduce the hysteresis, a pulse width modulation (PWM) waveform is often used in active matrix and passive EWDs. In high-resolution EWDs, a large number of pixel voltages need to be accurately controlled for playing images and videos. The driving waveform is a sequence of driving voltages controlling the pixel for the switching state. However, the trapped charge in the dielectric layer cannot be released by the PWM waveform; the oil backflow phenomenon still exists in EWDs. The stable display of multi-level grayscale images and videos is still a problem to be solved [23,24]. Many scholars have studied charge trapping and hysteresis in EWDs; alternate current (AC) waveforms and reset waveforms have been proposed to suppress oil backflow. However, aqueous solutions are often used as polar liquids for EWDs, and the indium tin oxide can be oxidized and reduced to a metal element by the reverse voltage of the AC waveform [25]. Generally, high-resolution EWDs are driven by the PWM waveform, which divide a frame into several subframes to display gray scales. Then, a driving waveform of the four-level grayscale was proposed by Yi [26], which was constructed using four sub-frames and a reset sub-frame. Since the charging time was much longer than the discharging time, oil backflow slowed. A period reset waveform with extra structures in the substrate was proposed by Choi to maintain the state of oil [27]; the extra structures included a notch electrode, a reset signal line, and a pixel electrode. However, only the trapped charges in the extra structure could be released by the reset signal. In 2016, a driving waveform which was constructed using seven sub-frames and a dynamic reset frame was proposed by Luo to display 16 grayscales in EWDs [28]. The increase in the number of sub-frames made the display flicker, and the shortened reset time made it take less time to discharge the charge.

In summary, these driving waveforms concern how to insert reset waveforms to overcome charge trapping. In this paper, a multilevel grayscale driving system with separate reset waveforms is proposed to suppress the oil backflow in high-resolution EWDs. The proposed reset waveform can be dynamically controlled by display content, which can avoid the increases in response time and periodic flickers. In addition, the separate reset waveform reduces the difficulty of waveform design, which makes this technique promising for display applications.

## 2. Principles of EWDs

**Figure 1:**
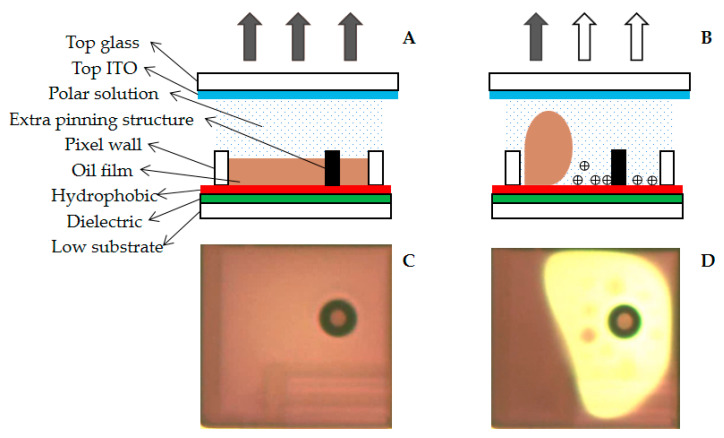
Structure of an EWD and the top view of an EWD under a microscope. (**A**) When no voltage is applied, the ratio of oil to water on the surface of the dielectric layer is 1:0 so that the pixel reflects the color of oil. (**B**) When a voltage is applied, the ratio of oil to water on the surface of the dielectric layer is 1:4 so that pixels mainly reflect the color of the reflective panel. (**C**) The top view of an EWD without a voltage applied; the oil is spread in the pixel. (**D**) The top view of an EWD with an applied voltage; the oil is pushed into a corner.

The principle of EWDs is to control the wetting force of the solid–liquid interface by the external electric field to promote the movement of oil on the solid interface. The pixel switching function is realized by controlling the spread and contraction of oil in a pixel. The working principle and pixel structure of EWDs are shown in Figure 1. The structure of an EWD pixel is shown in Figure 1A, including a top glass, polar solution, a hydrophobic layer, pixel wall, oil, an extra pinning structure, a dielectric layer, a low substrate, and a reflective panel [29,30]. ITO glass with high transmittance is used as the top plate. A thin film transistor (TFT) substrate is used as the lower substrate. As shown in Figure 1B, the reflectivity of the EWD is affected by the lower substrate, the top plate, and the dielectric layer, and the ratio of the TFT area to the pixel area also affects the reflectivity. When no voltage is applied, the dielectric layer is lipophilic and hydrophobic so that the oil spreads in the pixel. The pixel presents the color of the oil, as shown in Figure 1C. When a voltage is applied to an EWD, the dielectric layer becomes hydrophilic. The oil is pushed into the corners of a pixel. At this time, the pixel presents the color of the reflective panel, as shown in Figure 1D. The proportion of oil and water on the surface of the dielectric layer is determined by the balance of the three-phase contact line [31]. The three-phase contact line is formed by the interfacial force among the oil, water, and the dielectric layer, and it can be modified when a voltage is applied to the system. The Lippmann–Young equation is the relationship between the liquid and solid contact angle and the driving voltage, as shown in Equation (1).

(1) $$\cos\theta \left(V\right)=\frac{\sigma_{do}-\sigma_{dw}\left(V\right)}{\sigma_{ow}}=\frac{\sigma_{do}-\sigma_{dw}\left(0\right)+\frac{\varepsilon_{0}\varepsilon_{d}}{2d_{t}}V^{2}}{\sigma_{ow}}=cos\theta_{0}+\frac{\varepsilon_{0}\varepsilon_{d}}{2d_{t}\sigma_{ow}}V^{2}$$

Where *σ* refers to surface/interfacial tensions, *d* is the dielectric, *w* is water, *o* is oil, *θ*_0_ is the initial contact angle when voltage is not applied, *θ*(*V*) is the contact angle when voltage *V* is applied, *ε*_0_ is the vacuum dielectric constant, and *ε_d_* and *d_t_* are the relative dielectric constant and thickness of the insulating layer, respectively.

## 3. The Mechanism of Active Matrix EWDs

**Figure 2:**
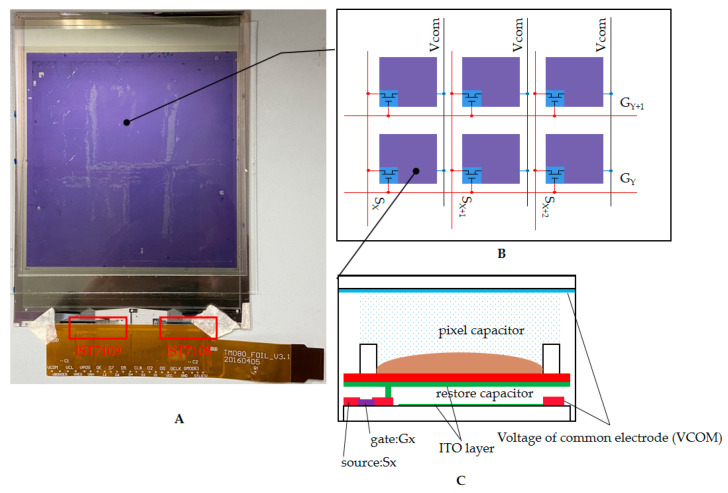
An active matrix EWD panel. (**A**) The appearance of the EWD module. The voltage of each pixel is precisely controlled by a scanning sequence composed of source and gate chips. (**B**) Equivalent circuit of a TFT substrate. Each pixel is controlled by a TFT for charging and discharging. The time-division multiplexing of the source signal is realized by the TFT array. (**C**) Cross-section views of a pixel and a TFT substrate. The voltage of the storage capacitor is controlled by the TFT so that continuous energy can be provided by the storage capacitor for the oil to maintain the state during the row and column scanning.

A TFT substrate is the basic structure of an active matrix EWD. As shown in Figure 2A, the EWD panel is driven by two cascaded chips (IST7109 and IST7108). An equivalent schematic diagram of the EWD panel is shown in Figure 2B. G_Y_ is a control signal that can control gates of TFTs of all pixels on a Y row. S_X_ is a signal that can supply power to sources of all TFTs on a column X. A common electrode is connected to the top plate so that the voltage of the storage capacitor is consistent with the pixel driving voltage. The storage capacitor can maintain the driving voltage after the TFT is switched off. The cross-sectional structure of the pixel is shown in Figure 2C. When the TFT is turned on, the voltage is directly transmitted to the ITO layer through the transparent electrode. The drive of a high-resolution EWD is usually realized by time-division multiplexing, which combines all S_X_ signals into a one-series data stream. In TFT parameters, the maximum charging time of a pixel can be obtained according to the defined refresh rate and resolution. During the frame-to-frame interval time and the scan line closing time in advance, pixels are not charged, and the actual maximum charging time is as shown in Equation (2).

(2) $$dt_{charge}=\frac{1}{Freq\times dot\_y}-Sync-Delay$$

Where *Freq* is the refresh rate, *dot*_*y* is the number of gate lines, *Sync* is the interval time between frames, and *Delay* is the time taken to turn off. When the gate of the TFT is opened, the storage capacitor is charged. Since the pixel voltage can never reach the charging voltage value, the pixel voltage only needs to reach 99.8% of the charging voltage in the traditional TFT design, as shown in Equation (3).

(3) $$V_{p}/V_{d}\geq 99.8\%$$

Where *V_p_* is the pixel voltage and *V_d_* is the charging voltage. By estimating the magnitude of the pixel capacitance, the range of the TFT on-state resistance can be obtained, as shown in Equation (4).

(4) $$R_{on}=\frac{-dt_{charge}}{C_{p}\cdot \ln(1-V_{p}/V_{d})}$$

where *C_p_* is pixel capacitance and *R_on_* is the TFT on-state resistance. When the gate of the TFT is switched off, the charge leakage in the pixel capacitor is caused by the off-state resistance of TFTs. At this time, the voltage drop caused by the leakage current cannot be lower than the voltage of the adjacent grayscale. When the number of grayscales is designed as *N*, the initial pixel voltage is dropped by discharge, which must satisfy Equation (5).

(5) $$V_{p}^{\prime }/V_{p}\geq 1/2N$$

where $V_{p}^{\prime }$ is the initial voltage and *V_p_* is the dropped voltage. Therefore, the off-state resistance of the TFT can be obtained, as in Equation (6).

(6) $$R_{off}=\frac{dt_{charge}-\frac{1}{Freq}}{C_{p}\times \ln\left(V_{p}^{\prime }/V_{p}\right)}=\frac{\frac{1}{Freq}-dt_{charge}}{C_{p}\times \ln\left(2N\right)}$$

where *R_off_* is the off-state resistance of the TFT. According to Equations (7) and (8), the ratio of the channel width to the channel length of a TFT is obtained.

(7) $$I_{ds}=\mu \cdot C_{ox}\cdot \frac{W}{L}\left(V_{gs}-V_{th}-\frac{1}{2}V_{ds}\right)V_{ds}$$

(8) $$I_{ds}=\frac{1}{2}\mu \cdot C_{ox}\cdot \frac{W}{L}{\left(V_{gs}-V_{th}\right)}^{2}$$

where *μ* is the electron mobility, *C_ox_* is the capacitance per unit area of the metal insulator semiconductor (MIS) structure, *V_ds_* is the drain-source voltage, *V_gs_* is the gate-source voltage, and *V_th_* is the threshold voltage.

When a voltage is applied to a pixel for a long time, as shown in Figure 1B, the oil cannot maintain the open state. This phenomenon is called oil backflow [32,33]. Researchers found that when a voltage is applied, the surface of the dielectric layer accumulates many charges [34].

**Figure 3:**
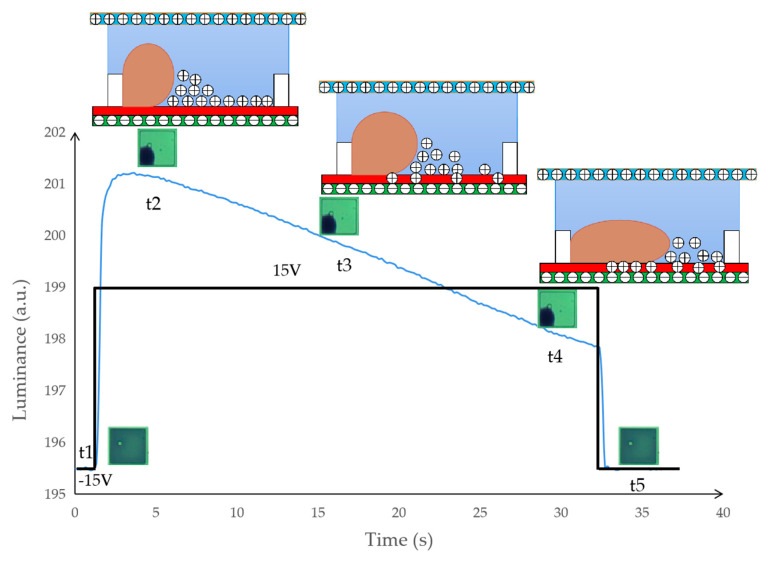
The illustration of oil backflow behavior in EWDs. The intensity curve of the reflected light when the pixel is turned on continuously for 30 s. The pixel is turned off at t1, the pixel is turned on at t2, the charge is embedded in the dielectric layer at t3, and the oil reaches an equilibrium state at t4.

Charges are gradually embedded into the dielectric layer through the force of the electric field, which can reduce the intensity of the electric field inside the pixel. The location of the three-phase contact line cannot be maintained with the decrease in electric field intensity. As shown in Figure 3, the oil is spread in the pixel without applying voltage at t1. At t2, 30 V DC is applied to the pixel and the oil is pushed to a corner. At the same time, charges begin to be embedded in the dielectric layer. At t3, the driving voltage is maintained at 30 V. Due to the effect of the embedded charge, the electric field force on the oil is reduced. Then, the oil reaches the equilibrium state again. At t4, the driving voltage is kept constant. The amount of electric charge retained by the dielectric layer is limited so the oil backflow can be stopped.

## 4. Design of Reset Waveform Using Overdriving Voltage

**Figure 4:**
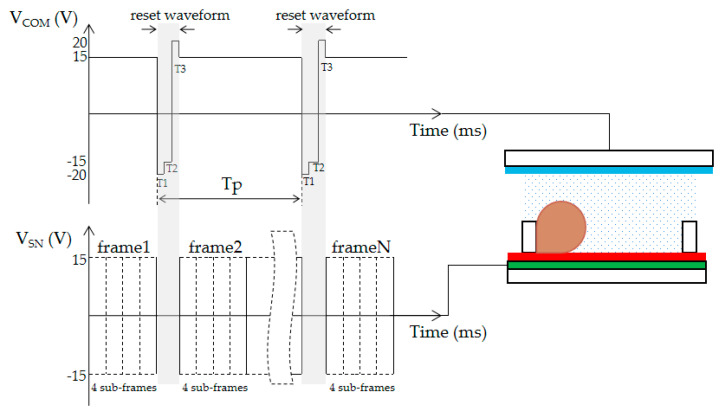
Reset waveform generated by the VCOM. The output of the reset waveform is controlled by the frame detection module in the system. The reset period is dynamically adjusted according to the displayed content.

Reset waveforms can be effectively used to overcome oil backflow. When a reset waveform is inserted into the driving waveform, the driving time can be extended in the traditional driving waveform. It can cause obvious flickers of EWDs and increase the response time. Therefore, an external reset by adjusting the voltage of common electrode (VCOM) voltage was designed in our system. Without inserting the reset waveform in the frame, the instantaneous reverse driving of the pixel can be achieved by adjusting the VCOM. The embedded charge can be released quickly with the overdriving in the reset waveform. After oil backflow is overcome, the grayscale can be maintained in EWDs. The dynamical reset waveform with overdriving voltage is shown in Figure 4. There are four voltages used in a reset waveform: −15 V, +15 V, −20 V, and +20 V. As shown in Figure 4, V_COM_ is an alternating current driving voltage that is connected to an EWD’s entire panel common electrode, V_Sn_ is the TFT source driving signal, and Tp is the reset cycle time; a frame is composed of four sub-frames. The response time of the oil is accelerated by a short time of high-voltage driving so that the oil can be pushed to a previous state quickly after a reset waveform.

The reset waveform is composed of a charge-releasing stage and oil moving back stage. T1 and T2 are two phases in the charge releasing stage, which are used to release the trapped charges and keep the oil active. In the charge releasing stage, the T1 phase is set as the overdriving voltage and the T2 phase is set as the reverse voltage. The T3 phase is an oil moving back stage, which is used to push the oil back to the previous state.

## 5. Results and Discussion

In order to study the influence of driving waveforms on the reflectivity of an active matrix EWD, a driving system was designed for active matrix EWDs, and the reflectivity range of the traditional PWM waveform and the dynamic reset waveform proposed in this paper were tested. The TFT substrate used in active matrix EWDs was co-developed by South China Normal University and Tianma Microelectronics Co. Ltd. (Shanghai, China). Its resolution is 1024 × 768. The pixel voltage is charged by the source chip. The gate of TFTs is controlled by the gate chip. Then, the precise voltage control of each pixel can be realized in the active matrix EWD. The source and gate functions are integrated into an IST7109 chip, which can control a 512 × 384 active matrix. Its source function includes a 512 bit long, 2 bit wide serial input register and a 2 bit encoder. The 2 bit encoder is used to choose the output voltage between 20, 0, and −20 V. IST7108 has the same function as IST7109. The IST7108 is the cascade chip of IST7109. The two chips are bound to a TFT substrate by chip on glass (COG) technology. The connection between the chip and the driving circuit board is completed through a flexible cable.

**Figure 5:**
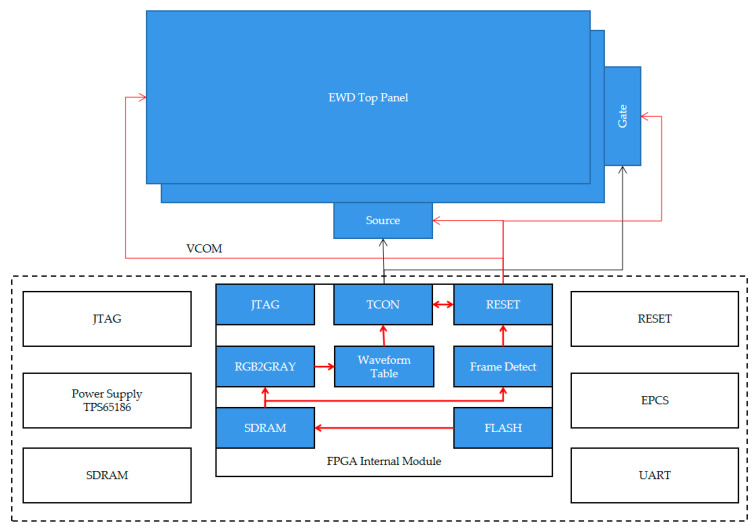
The block diagram of the driving board and the function module of a FPGA. After the system is powered on, RGB image data are read from FLASH to SDRAM and the image data are converted into gray tone data by the RGB2GRAY module. Then, the gray tone data are used to find the waveform in the waveform table and output it to a FPGA internal module. Finally, the source and gate chips are controlled for display. Pixels can be driven by the final waveform with the control of the source and gate chips. The data frame buffer in SDRAM is detected by the frame detection module to control the dynamic output of the reset waveform.

The driving system structure used in this study is shown in Figure 5. A filed program gate array (FPGA) in the system is mainly responsible for the output of the driving waveform and the dynamic reset waveform. The dynamic reset waveform and grayscale waveform are output independently so the driving time of the grayscale waveform is shortened. Then, movement of oil in the pixel can be controlled more precisely by the grayscale waveform. The joint test action group (JTAG) interface of FPGAs is used to download the complied code with a USB blaster cable. The image data are stored in an erasable, programmable flash chip. a synchronous dynamic random-access memory (SDRAM) is responsible for image data caches. A TPS65186 (Texas Instruments, American) is used as a power supply IC to generate all 0 V, −15 V, +15 V, −20 V, and +20 V voltages in one chip as a power management solution for EWDs. The gate is responsible for controlling the pixel switch of each column, and the source provides voltage for each row of pixels in the pixel matrix. A timing controller (TCON) is used to control the cooperative work of gate and source chips. Since the driving voltage, which is required for oil movement, is 30 V, the liquid crystal driver chip cannot meet the requirements for controlling EWDs. Therefore, IST7109 was chosen as the source chip; it can output 0 V, +15 V and −15 V. In order to achieve the driving voltage required by EWDs, the top plate is connected to +15 V, which can output driving voltages of 0 V, 15 V, and 30 V.

Related materials of TFT substrates that are used in our system are shown in Table 1. To enable comparison with the traditional PWM method, an optical colorimeter arg-45, developed by Admesy in the Netherlands, was used to obtain the reflected luminance of the EWD and a camera was used to record the video and image. The driving system was powered by a power adapter. The specifications of EWDs are shown in Table 2.

**Figure 6:**
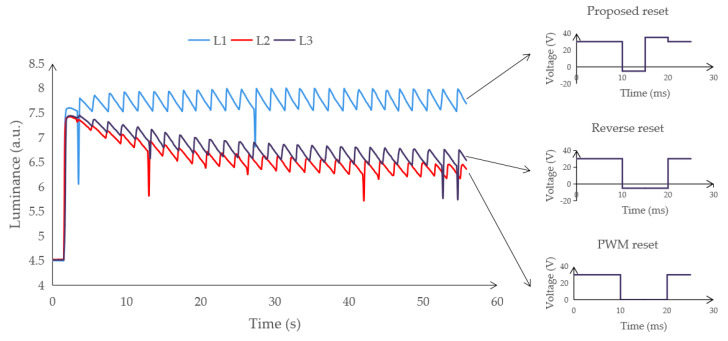
The influence of three different reset waveforms on oil backflow. A negative voltage (−5 V) is contained in the reverse reset waveform. A zero voltage is contained in the PWM reset waveform.

The colorimeter can emit light at an angle of 45° to irradiate pixels in an area. After the light is transmitted by the EWD panel, it can be reflected by the reflective panel and passed through the EWD panel again. Then, the intensity of the reflected light can be obtained by the colorimeter. In this experiment, the influence of different reset waveforms on oil backflow was compared, as shown in Figure 6. An EWD was driven by the same voltage (30 V) with a different reset waveform. Based on the experimental data, the proposed reset waveform more effectively maintains the oil state.

**Figure 7:**
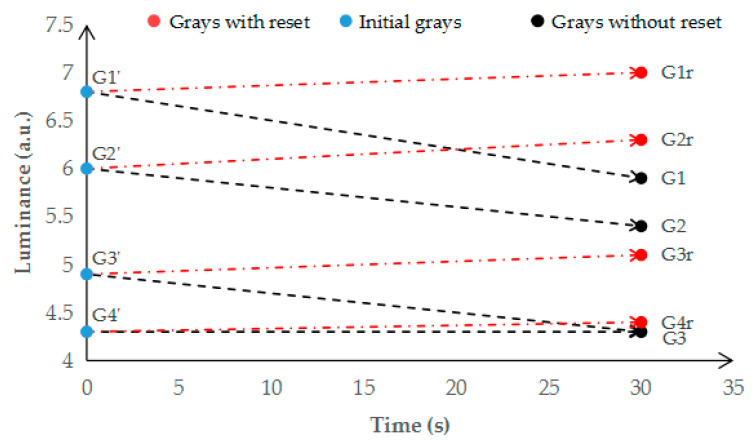
Grayscales affected by oil backflow. The luminance of four grayscales decreased after 30 s. After 30 s, the luminance of G3′ was the same as G4′. The initial luminance of grayscales was G1′, G2′, G3′, and G4′. After 30 s, the grayscales without the reset waveform ere changed to G1, G2, and G3, and grayscales with the reset waveform changed to G1r, G2r, G3r, and G4r.

The influence of grayscale driven by the reset waveform is shown in Figure 7. The luminance of grayscales G1′, G2′, G3′, and G4′ cannot be maintained without the reset waveform. After 30 s, four grayscales are decreased to three gray scales G1, G2, and G3. However, the grayscales of G1r, G2r, G3r, and G4r can be maintained by the reset waveform. The fluctuation of brightness was controlled within 4%.

**Figure 8:**
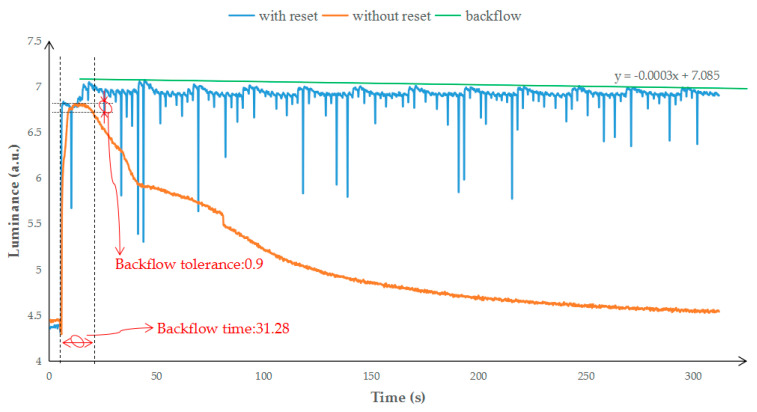
The relationship between the reflected brightness and time for different driving methods. The “with reset” curve is the brightness data, which are driven with the reset waveform. The “without reset” curve is the brightness data, which are driven with the traditional driving waveform. The linear backflow is used to evaluate the oil backflow speed.

In the reset waveform and the PWM waveform, the reflected brightness curves are shown in Figure 8. When there is no reset waveform, the oil state in a pixel cannot be maintained beyond oil backflow time. The oil backflow start time is the time when the reflectivity decreases from the maximum value to 90% of the maximum value. The oil backflow start time without the reset waveform was 31.28 s. When the reset waveform was used, the response curve is with reset. The reflected brightness oscillated periodically, and the oscillation period was about 24 s.

In order to analyze the influence of oil backflow, we used the method of numerical fitting for statistical analysis. First, the maximum points in each oscillation period were extracted as the local maximum data in the with reset curve. Second, the maximum point (44.39, 7.07007) and minimum point (196.19, 6.98932) were found in the local maximum data. Third, the maximum point and minimum point were used to represent the worst-case oil backflow percentage (1.14%) after a reset waveform; a straight line was used to simply predict the oil backflow situation. Finally, according to the calculation of the straight line, the oil backflow start time was 23,616 s. The oil backflow time was extended by 761 times compared with PWM driving.

**Figure 9:**
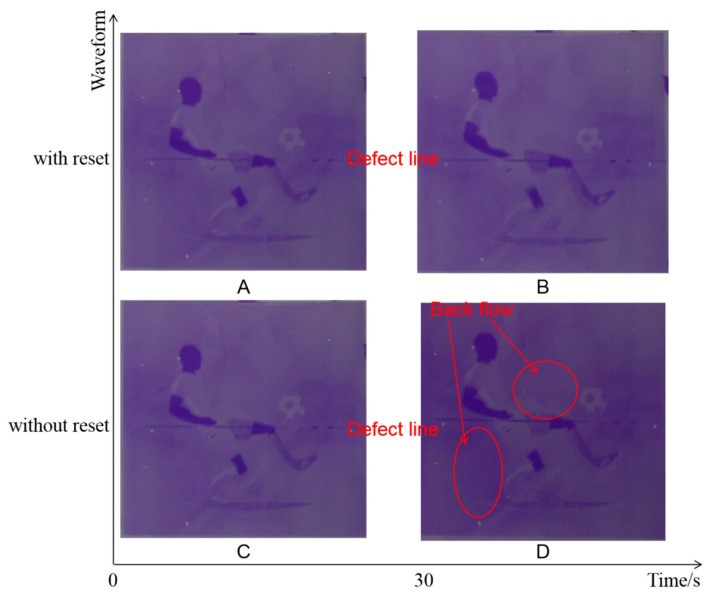
Comparison of static images with different driving waveforms. The ordinate indicates the driving waveform used in the experiment, and the abscissa indicates time. At 0 s, image (**A**) was recorded with the reset waveform and image (**C**) was recorded without the reset waveform. After 30 s, image (**B**) was recorded with the reset waveform and image (**D**) was recorded without the reset waveform. There is an uncontrollable line in the middle of the screen, which is a defect in the gate chip binding.

The static image effect comparison among different driving waveforms is shown in Figure 9. When the EWD was switched on, a camera was used to record the EWD screen. After 30 s, the camera was used to take another recording. The defect line on the screen is a chip bonding defect. As a result, the display quality was improved intuitively by the reset waveform.

The system successfully realizes the reset function of EWDs with 1024 × 768 resolution. The oil backflow of the static display is effectively suppressed, and the frame loss and obvious flicker caused by the insertion of the reset waveform are avoided at the same time, providing a new driving technology foundation for EWDs.

## 6. Conclusions

In this paper, the separated reset waveform composed of +15, −15, +20, and −20 voltages was presented. Compared with the reset waveform in PWM, the reset waveform has more output levels and can be dynamically inserted between frames to reduce the effective driving time extension caused by adding a reset time slot in each frame. The reset waveform can be controlled independently without changing the structure of the TFT substrate. The experimental data confirmed that the oil backflow can be effectively suppressed by the driving method with a reset waveform separation. By comparing the optical data, the oil backflow time is extended by 761 times compared with PWM driving. The separated reset driving waveform demonstrates the performance of static image, which greatly improves the display performance and reading comfort of the EWD. This design route can be applied for multi-color, high-resolution EWDs in the future.

## Figures and Tables

**Table 1:** The material parameters of a TFT substrate.

Metal of Gate	Semiconductor Material	Drain/Source Metal	Transparent Electrode
MoW	A-Si	Al-Nd	ITO

**Table 2:** Specifications of an 8 inch EWD panel.

Pixel Number	Pixel Wall Width	Pixel Wall Thickness	Hydrophobic Layer	Top ITO Thickness
1024 × 768	12 μm	6 μm	1 μm	25 μm

## Data Availability

The data presented in this study are available in Section 5.
